# Supplementary figures and images for: CD9 co-operation with syndecan-1 is required for a major staphylococcal adhesion pathway
Source: mBio. 2023 Jul 24;14(4):e01482-23. doi: 10.1128/mbio.01482-23 (PMC10470606; doi:10.1128/mbio.01482-23)

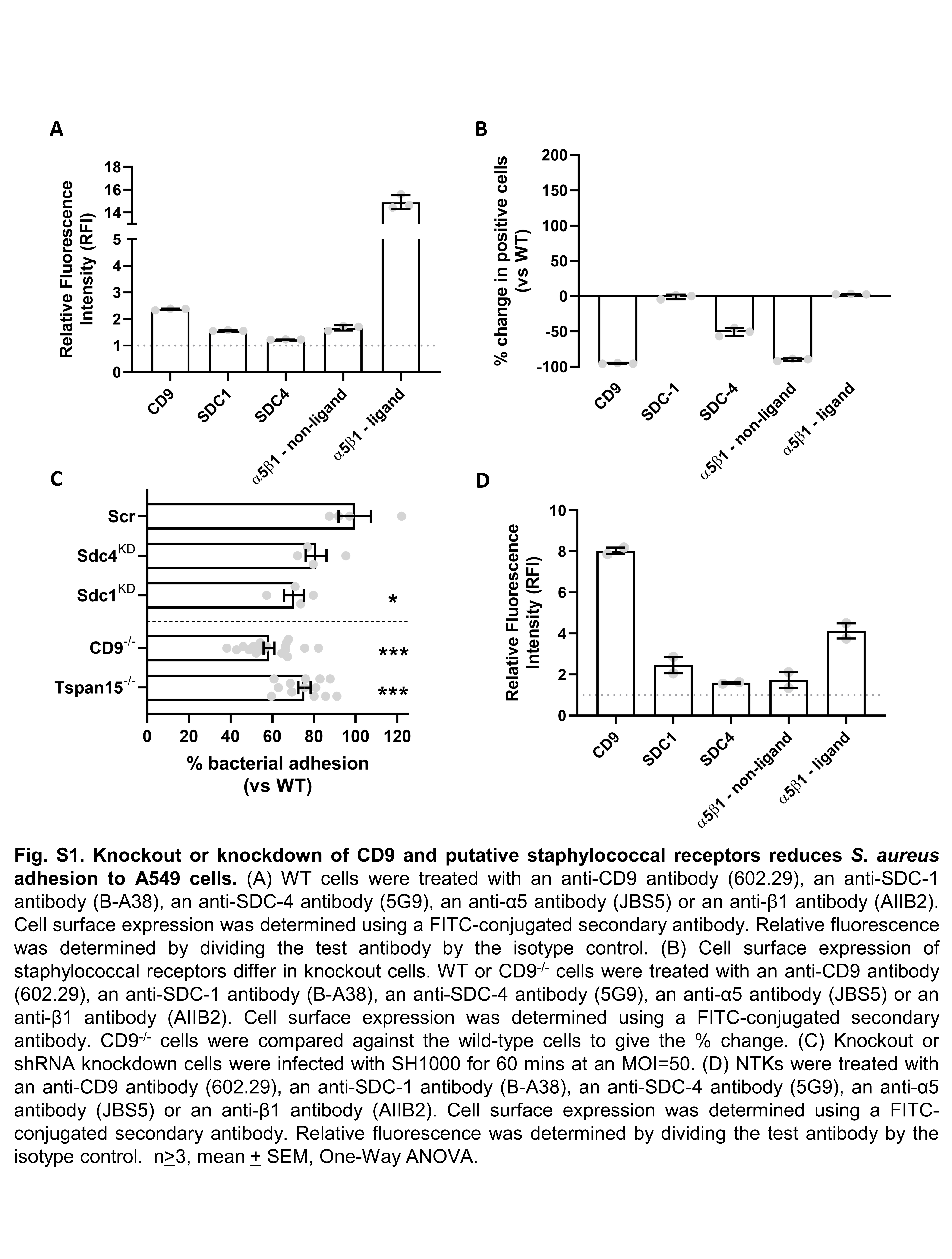

Supplement: Fig. S1 — Knockout or knockdown of CD9 and putative staphylococcal receptors reduces S. aureus adhesion to A549 cells. [file mbio.01482-23-s0001.tif]

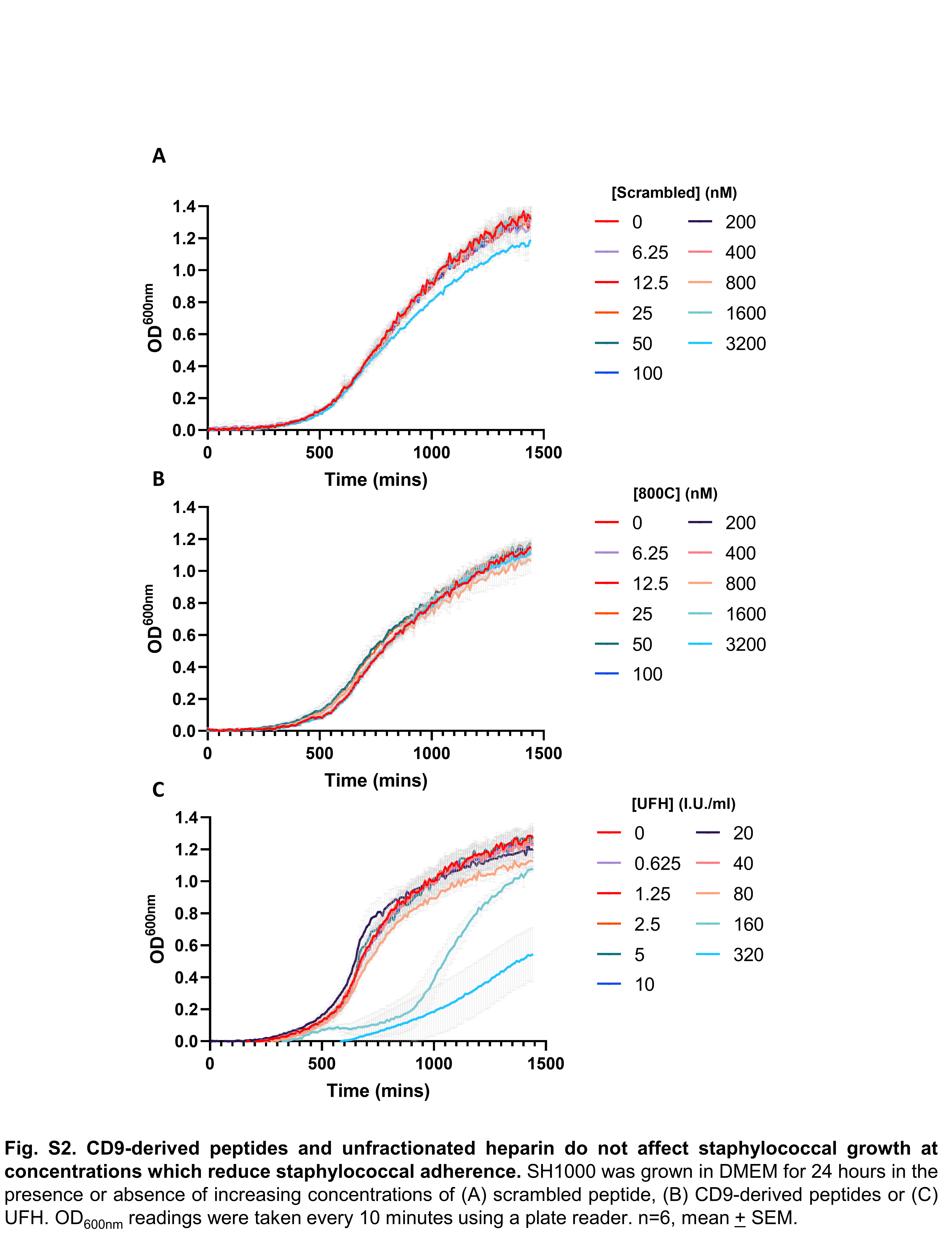

Supplement: Fig. S2 — CD9-derived peptides and unfractionated heparin do not affect staphylococcal growth at concentrations which reduce staphylococcal adherence. [file mbio.01482-23-s0002.tif]

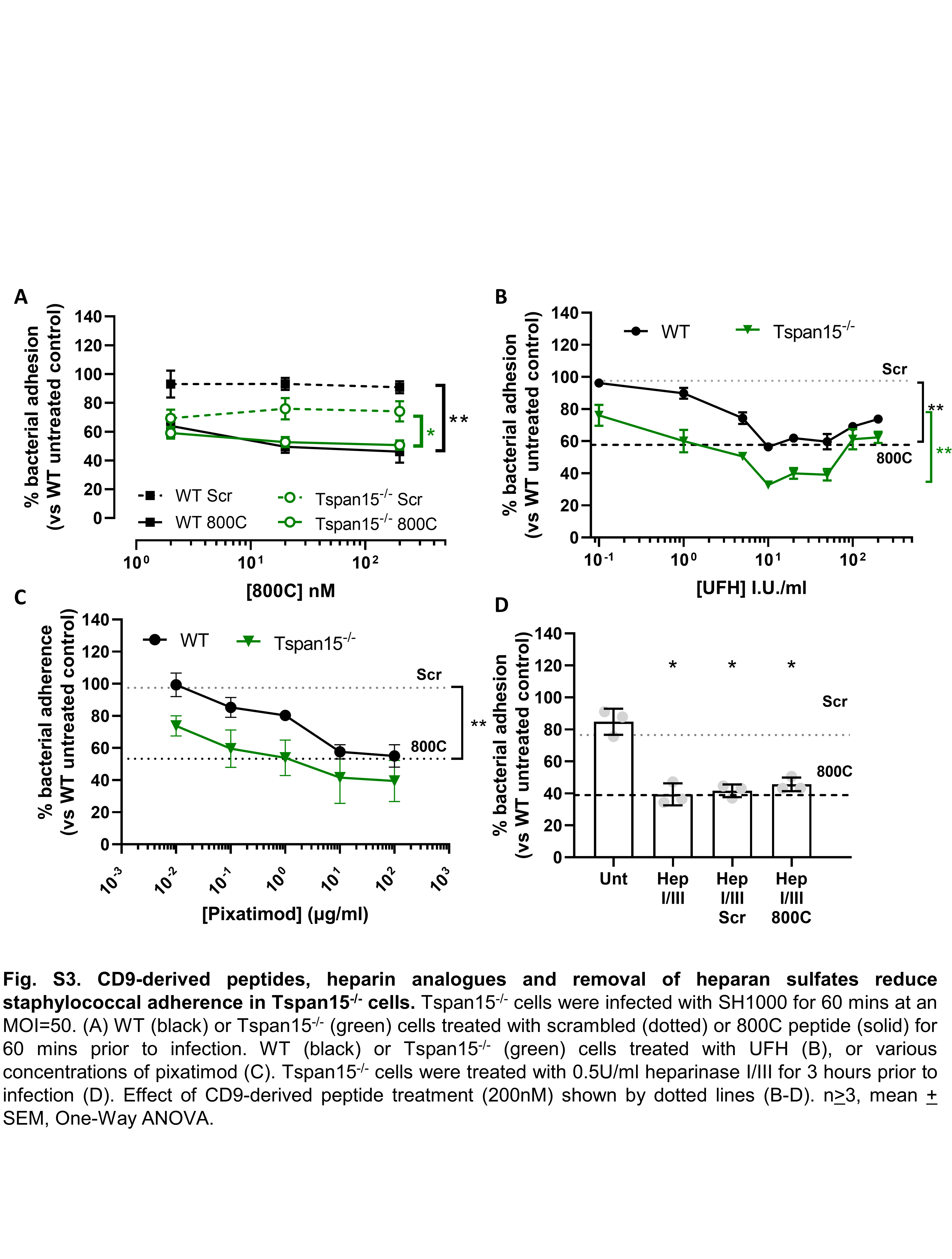

Supplement: Fig. S3 — CD9-derived peptides, heparin analogues and removal of heparan sulfates reduce staphylococcal adherence in Tspan15−/− cells. [file mbio.01482-23-s0003.tif]

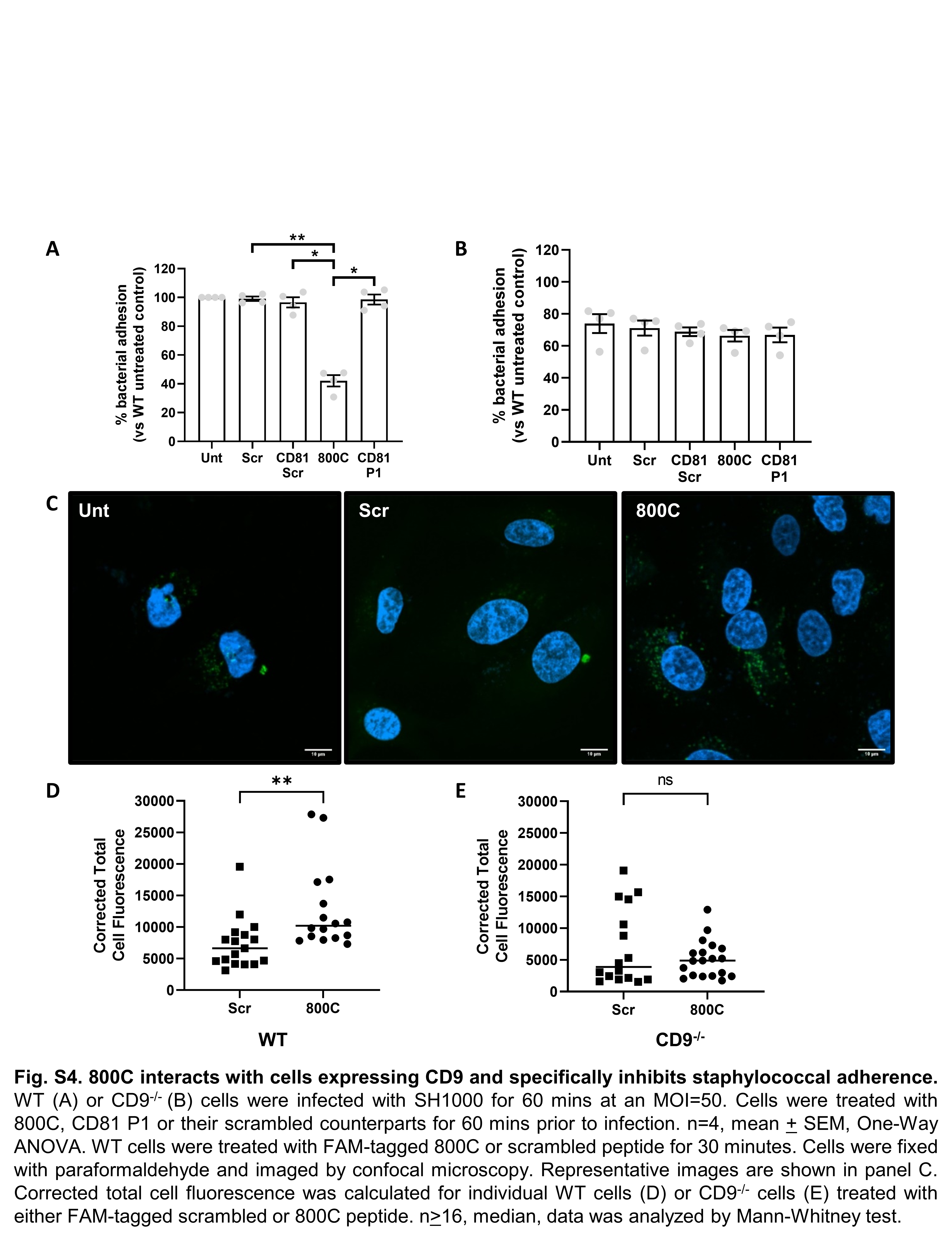

Supplement: Fig. S4 — 800C interacts with cells expressing CD9 and specifically inhibits staphylococcal adherence. [file mbio.01482-23-s0004.tif]

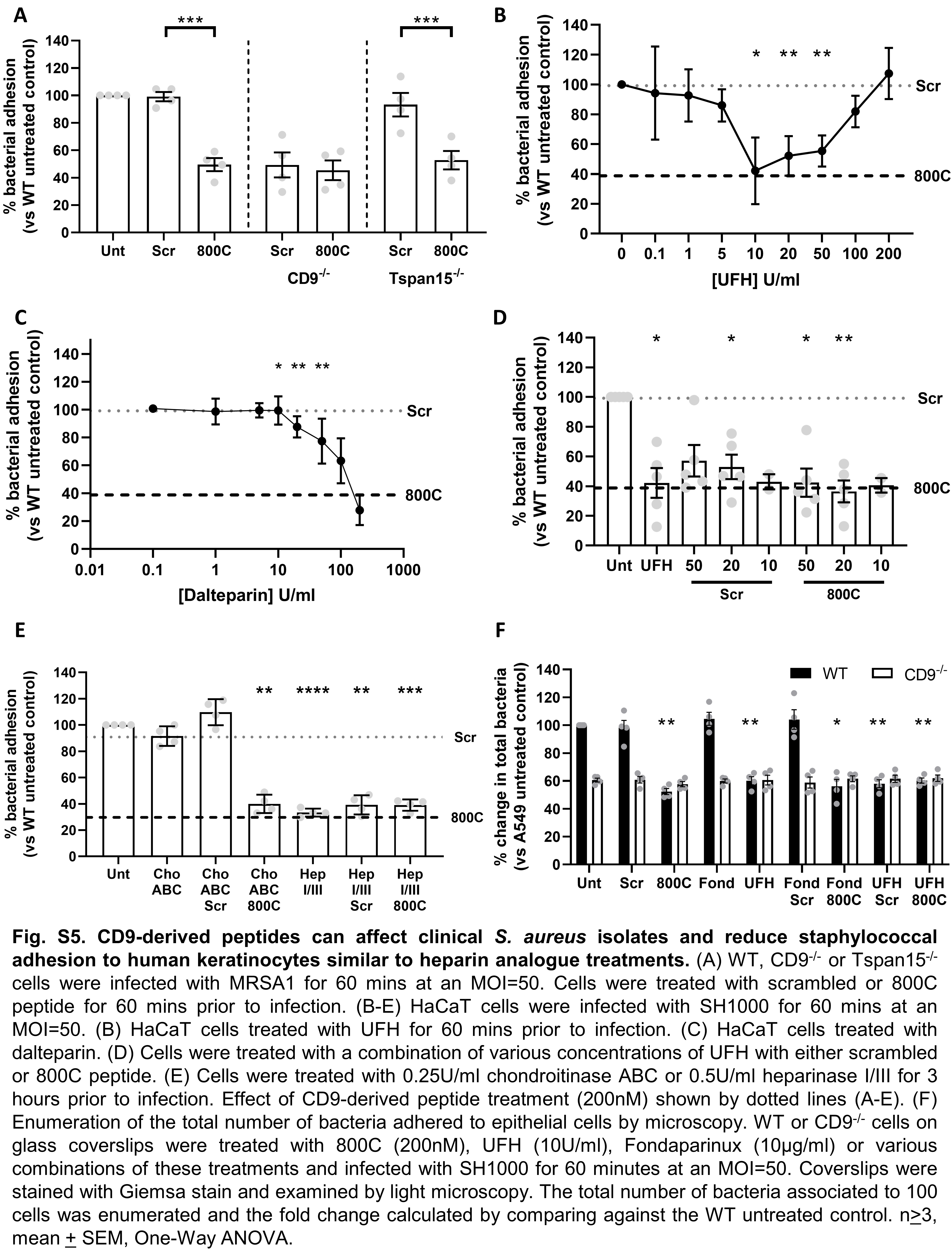

Supplement: Fig. S5 — CD9-derived peptides can affect clinical S. aureus isolates and reduce staphylococcal adhesion to human keratinocytes similar to heparin analogue treatments. [file mbio.01482-23-s0005.tif]

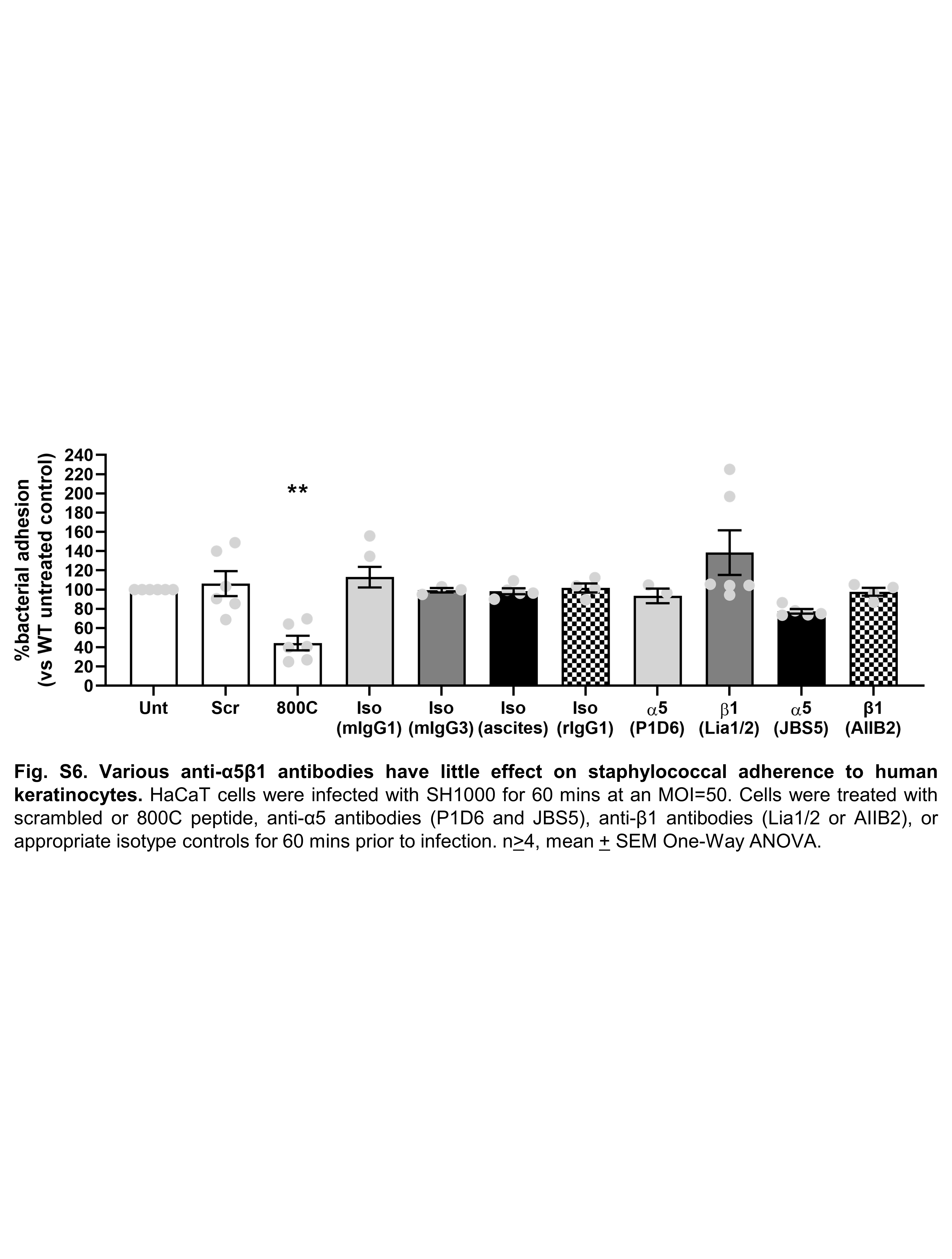

Supplement: Fig. S6 — Various anti-α5β1 antibodies have little effect on staphylococcal adherence to human keratinocytes. [file mbio.01482-23-s0006.tif]

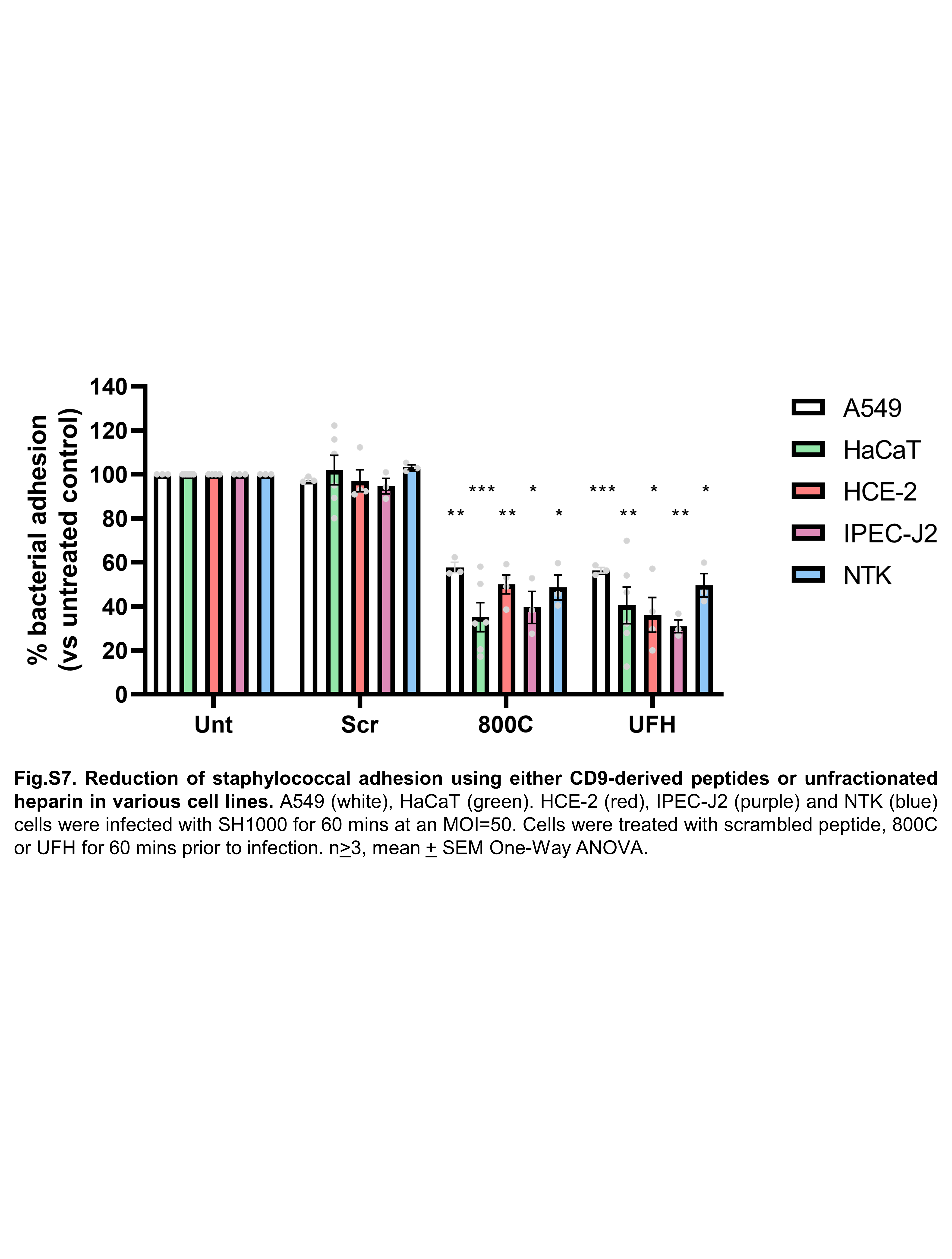

Supplement: Fig. S7 — Reduction of staphylococcal adhesion using either CD9-derived peptides or unfractionated heparin in various cell lines. [file mbio.01482-23-s0007.tif]

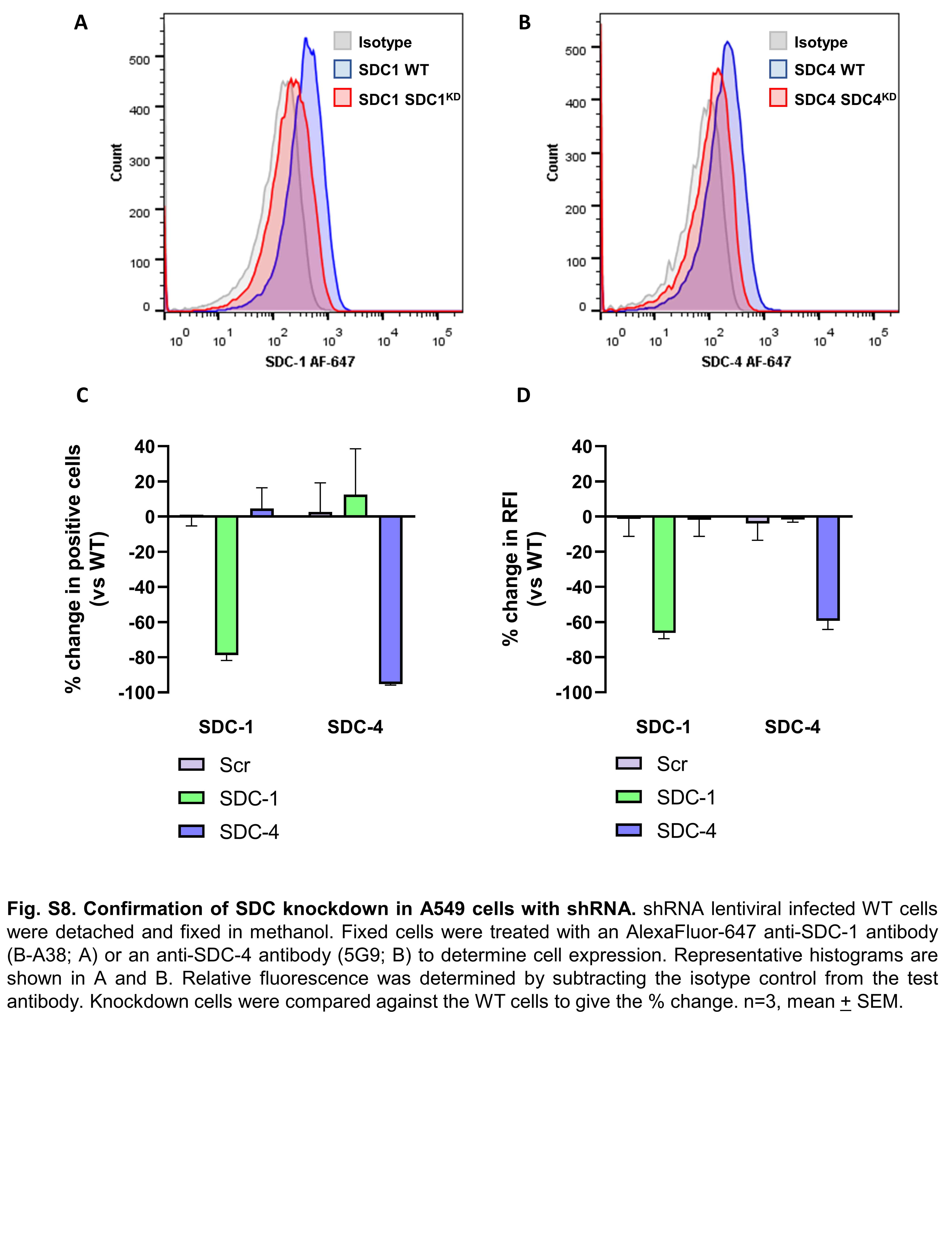

Supplement: Fig. S8 — Confirmation of SDC knockdown in A549 cells with shRNA. [file mbio.01482-23-s0008.tif]

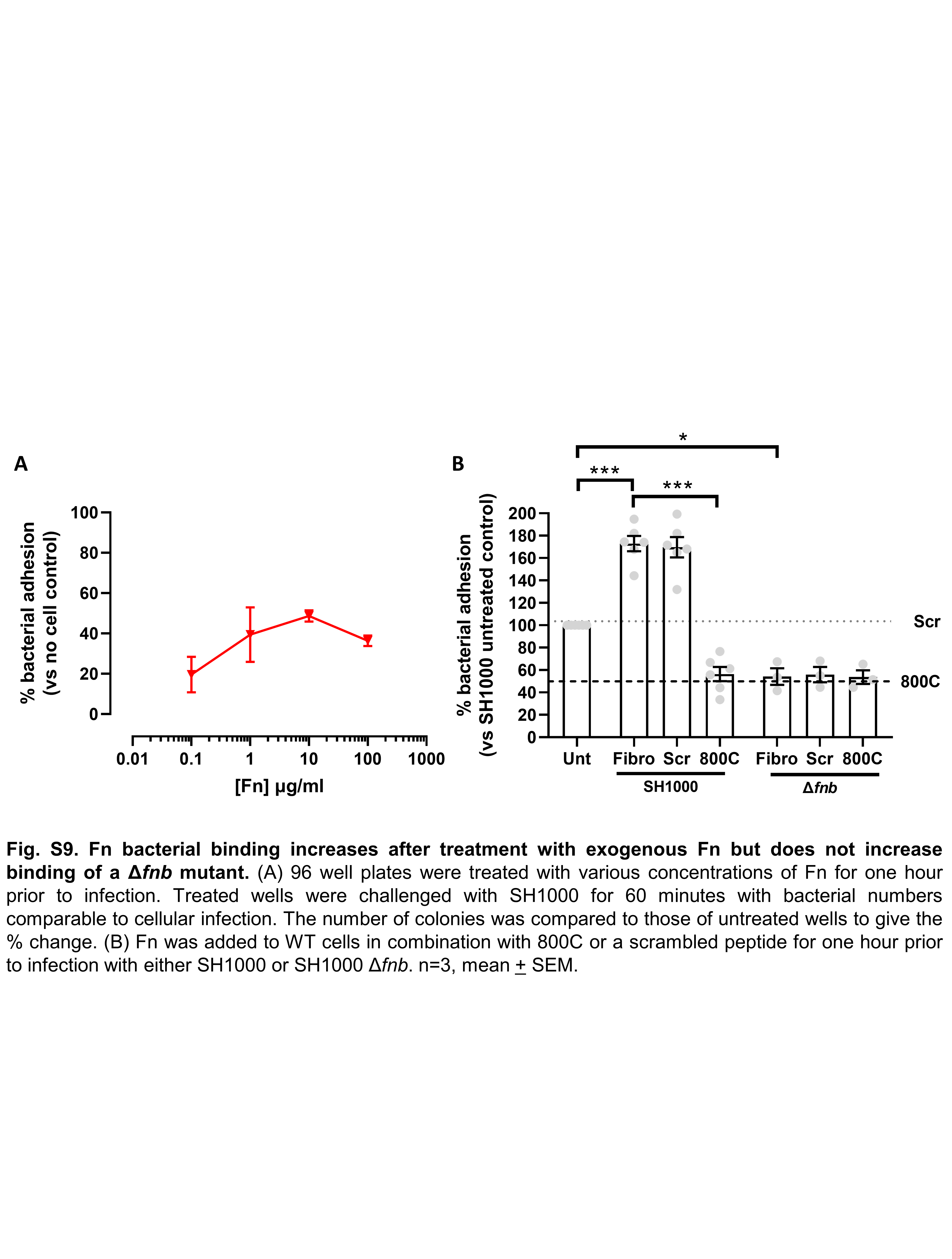

Supplement: Fig. S9 — Fn bacterial binding increases after treatment with exogenous Fn but does not increase binding of a Δfnb mutant. [file mbio.01482-23-s0009.tif]
